# Loss of *SNAI2* in Prostate Cancer Correlates With Clinical Response to Androgen Deprivation Therapy

**DOI:** 10.1200/PO.20.00337

**Published:** 2021-06-22

**Authors:** Marek Cmero, Natalie J. Kurganovs, Ryan Stuchbery, Patrick McCoy, Corrina Grima, Anne Ngyuen, Ken Chow, Stefano Mangiola, Geoff Macintyre, Nicholas Howard, Michael Kerger, Philip Dundee, Paul Ruljancich, David Clarke, Jeremy Grummet, Justin S. Peters, Anthony J. Costello, Sam Norden, Andrew Ryan, Phillip Parente, Christopher M. Hovens, Niall M. Corcoran

**Affiliations:** ^1^ Department of Surgery, University of Melbourne, Parkville, Victoria, Australia; ^2^ Division of Bioinformatics, Walter and Eliza Hall Institute, Parkville, Victoria, Australia; ^3^ Peter MacCallum Cancer Centre, Melbourne, Victoria, Australia; ^4^ Sir Peter MacCallum Department of Oncology, The University of Melbourne, Parkville, Victoria, Australia; ^5^ Department of Urology, Royal Melbourne Hospital, Parkville, Victoria, Australia; ^6^ Cancer Research UK Cambridge Institute, University of Cambridge, Cambridge, United Kingdom; ^7^ Department of Urology, Peninsula Health, Frankston, Victoria, Australia; ^8^ Department of Urology, Box Hill Hospital, Box Hill, Victoria, Australia; ^9^ Epworth Eastern Hospital, Box Hill, Victoria, Australia; ^10^ Department of Urology, Alfred Hospital, Prahan, Victoria, Australia; ^11^ Monash University, Clayton, Victoria, Australia; ^12^ TissuPath, Mount Waverly, Victoria, Australia; ^13^ Department of Medical Oncology, Box Hill Hospital, Box Hill, Victoria, Australia; ^14^ Victorian Comprehensive Cancer Centre, Melbourne, Victoria, Australia

## Abstract

**PURPOSE:**

Androgen receptor (AR) signaling is important in prostate cancer progression, and therapies that target this pathway have been the mainstay of treatment for advanced disease for over 70 years. Tumors eventually progress despite castration through a number of well-characterized mechanisms; however, little is known about what determines the magnitude of response to short-term pathway inhibition.

**METHODS:**

We evaluated a novel combination of AR-targeting therapies (degarelix, abiraterone, and bicalutamide) and noted that the objective patient response to therapy was highly variable. To investigate what was driving treatment resistance in poorly responding patients, as a secondary outcome we comprehensively characterized pre- and post-treatment samples using both whole-genome and RNA sequencing.

**RESULTS:**

We find that resistance following short-term treatment differs molecularly from typical progressive castration-resistant disease, associated with transcriptional reprogramming, to a transitional epithelial-to-mesenchymal transition (EMT) phenotype rather than an upregulation of AR signaling. Unexpectedly, tolerance to therapy appears to be the default state, with treatment response correlating with the prevalence of tumor cells deficient for *SNAI2*, a key regulator of EMT reprogramming.

**CONCLUSION:**

We show that EMT characterizes acutely resistant prostate tumors and that deletion of *SNAI2*, a key transcriptional regulator of EMT, correlates with clinical response.

## BACKGROUND

Prostate cancer is critically dependent on activation of the androgen receptor (AR), a ligand-activated transcription factor, for cancer cell growth and survival. As such, interference with AR activation by suppression of ligand synthesis (androgen deprivation therapy [ADT]) or direct receptor inhibition has been the backbone of treatment of advanced disease for many years.^1^ The majority of patients demonstrate a biochemical response as measured by a fall in serum prostate-specific antigen (PSA); however, the magnitude and duration of response are highly variable, with some patients progressing rapidly to castration resistance, whereas others experiencing prolonged periods of stable disease.^2^ Although the molecular mechanisms that drive progressive castration-resistant disease (castration-resistant prostate cancer [CRPC]) are reasonably well-characterized,^3-11^ what determines tumor cell survival in response to pathway inhibition in the short term is unknown. This is a significant knowledge gap in one of the world's most common cancers and an ongoing impediment to the development of predictive biomarkers as well as more effective treatment strategies.

**CONTEXT**

**Key Objective**
To investigate determinants of sensitivity to 6 months of neoadjuvant androgen receptor signaling inhibition in patients with high-risk prostate cancer.
**Knowledge Generated**
Treatment response is highly variable, with residual tumor characterized by transcriptional reprogramming to an intermediate epithelial-to-mesenchymal transition (EMT) state. Somatic loss of SNAI2, a key regulator of EMT, correlates with tumor response, suggesting that the inability of tumor cells to implement reprogramming is an important determinant of treatment sensitivity.
**Relevance**
These findings suggest that SNAI2 loss may be a useful biomarker in predicting response to androgen receptor signaling inhibitors and strategies targeting EMT may boost treatment efficacy.

To investigate this, we performed a phase II neoadjuvant study of profound androgen deprivation and receptor blockade (degarelix, abiraterone, and bicalutamide) for 6 months prior to prostatectomy in patients with prostate cancer with a high risk of disease recurrence and conducted an in-depth analysis of pre- and post-treatment specimens to identify molecular determinants of response to acute pathway inhibition as a secondary outcome.

## METHODS

### Clinical Trial

We performed an open-label nonrandomized neoadjuvant phase II study (ACTRN12612000772842) in men with prostate cancer with high-risk features (PSA > 20 ng/dL, predominant cancer Gleason pattern 4 or above, or clinical stage ≥ cT2c) with no evidence of bony metastatic disease (Data Supplement). Patients received degarelix 240/80 mg subcutaneously every 4 weeks, abiraterone acetate 500 mg orally daily titrating upward every 2 weeks by 250 mg to a final dose of 1,000 mg daily, bicalutamide 50 mg orally daily, and prednisolone 5 mg orally twice daily. Treatment was for 24 weeks (six cycles), and oral medications were continued up until the day of surgery. Sample size was calculated on the basis of an anticipated complete pathologic response (complete response [CR]) rate and used Simon's^12^ two-stage phase II trial design. On the basis of a historical CR rate of approximately 5% with conventional castration and the assumption that a CR rate of 25% would be of clinical interest for examination in a larger phase III trial setting, a maximum of 17 patients needed to be accrued to have 80% power to detect a significant difference at α = .05 level. From the initial nine patients, at least one observed CR was necessary to trigger recruitment to the second stage, with at least three CRs in the final cohort required to reject the null hypothesis. All study investigations had institutional review board approval.

All patients provided written informed consent before enrollment. All study interventions and investigations were approved by the Human Research Ethics Committee at Melbourne Health (HREC 2012.220).

### Fresh Tissue Banking

Frozen section confirmed fresh tumor and benign tissue were collected at the time of prostatectomy as previously described.^13^

### Pathologic Assessment

Specimens were routinely fixed and processed and tumor volume calculated as previously described.^14^ CR was defined as no identifiable residual tumor (ypT0). No response was defined as absence of histologic evidence of tumor involution. Partial response was defined as identifiable residual tumor with variable histologic evidence of tumor involution. Although not prespecified in the study Protocol, for consistency with recent reports, minimal residual disease was defined as a residual tumor volume ≤ 0.2 cc^15^ with no high-grade elements present (Gleason score ≤ 3 + 3). Tumor volumes were not corrected for cellularity.

### Experimental Methods

A detailed description of the experimental procedures used is provided in the Data Supplement.

## RESULTS

### Objective Responses to Profound AR Inhibitors Are Highly Variable and Not Associated With Established CRPC Drivers

**FIG 1.**
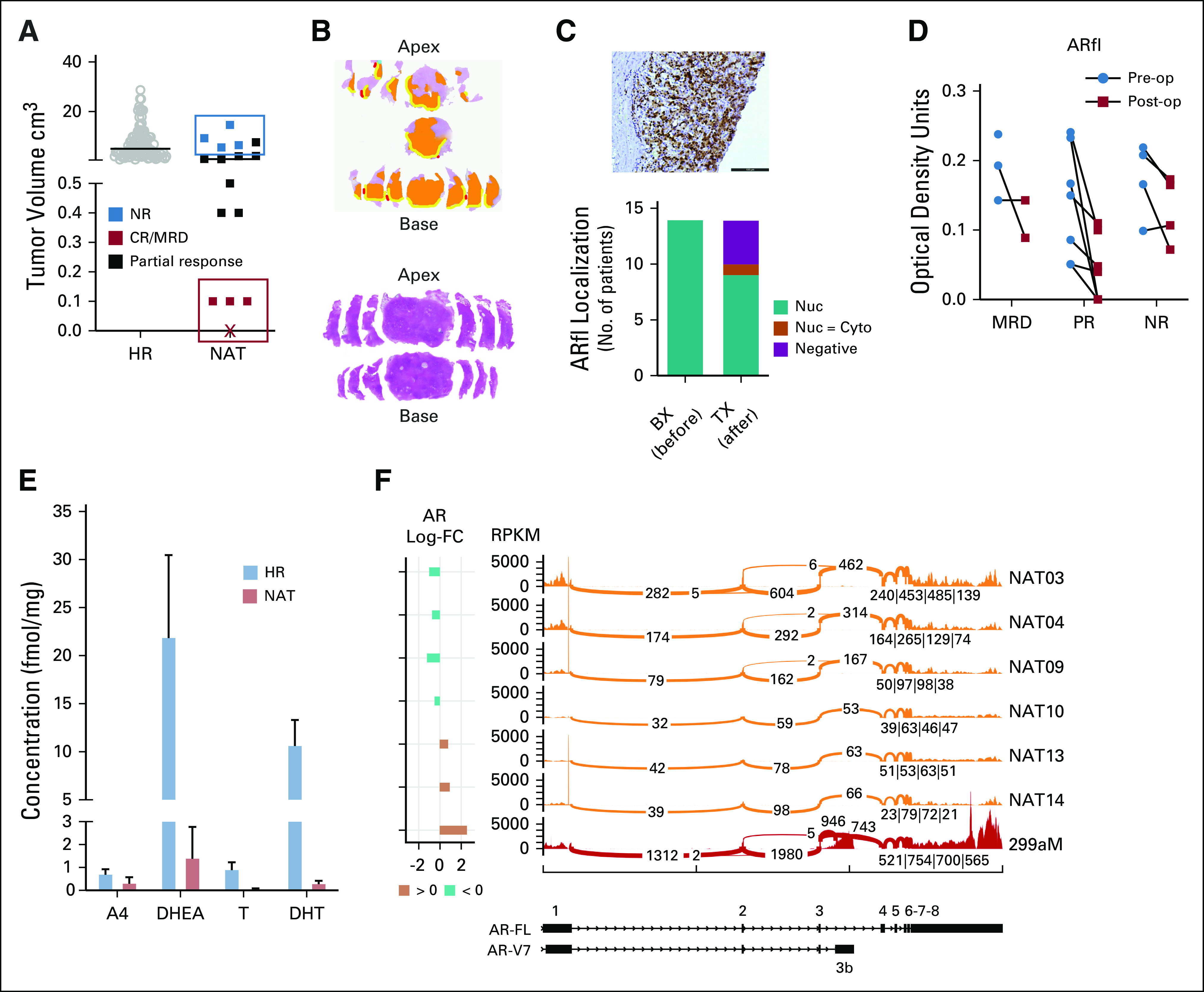
Common molecular drivers of breakthrough castration resistance are not detectable in persistent tumors. (A) Total tumor volume in patients treated with combination of profound androgen suppression and receptor blockade (termed NAT, n = 16) compared with a historical untreated cohort with similar pretreatment characteristics (termed HR, n = 101). The solid bar represents tumor volume median. Patient with a complete pathologic response is represented with an X. (B) Whole-mount section map of prostate from a patient with NR (upper panel) and CR (bottom panel). Tumor is marked in orange, with areas of extraprostatic extension highlighted in yellow. Red marks a positive margin. (C) Immunohistochemical staining for AR in nonresponder indicating nuclear staining. Bar equals 200 μm. Summary of AR location in before and after treatment specimens. (D) Patient-level data of mean optical density of quantitative digital analyses of AR staining categorized by treatment response. (E) Mean (+ SEM) concentration of signaling androgens in NAT prostate tissue (n = 12) as determined by high-performance liquid chromatography and tandem mass spectrometry with comparison with an untreated HR cohort (n = 10). (F) Sashimi plots for AR-FL and AR-V7 transcripts created using MISO. Exon regions for AR full length and AR-V7 are depicted graphically at the bottom, with a pileup of RNA-seq RPKM mapping to each exon in six NAT patients (orange tracks). The number of reads that span each exon boundary is shown. Comparison is made with a positive control (red track) with known AR-V7 upregulation. In all cases of residual tumor, the ratio of AR-V7 to full-length receptor expression was < 1%. A4, androstenedione; AR, androgen receptor; AR-FL, androgen receptor full length; AR-V7, androgen receptor splice variant 7; BX, biopsy; CR, complete response; Cyto, cytoplasmic; DHEA, dehydroepiandrosterone; DHT, dihydrotestosterone; FC, fold change; FL, full length; HR, high risk; MRD, minimal residual disease; MISO, mixture of isoforms; NAT, neoadjuvant therapy; NR, no response; Nuc, nuclear; PR, partial response; RPKM, reads per kilobase million; T, testosterone; TX, post treatment.

Seventeen patients were enrolled and completed neoadjuvant treatment, with one patient declining to proceed with prostatectomy. Despite all patients demonstrating a decrease in serum PSA > 95% (Data Supplement), objective pathologic responses were highly variable (Figs 1A and 1B). Four of 16 evaluable patients treated had sensitive cancers, with no remaining tumor (n = 1) or minimal residual disease (n = 3) identified in the prostatectomy specimen. In contrast, an identical number of patients had no measurable treatment effect observed. The remaining patients (n = 8) had partial tumor responses, with significant volumes of residual tumor but histologic evidence of at least some tumor regression. Heterogeneity in tumor response could not be explained by differences in the pretreatment disease characteristics of the patient groups (Data Supplement), and there was no correlation between PSA nadir and residual tumor volume (Data Supplement). The risk of disease recurrence, however, was closely linked to the extent of tumor regression (Data Supplement).

Reactivation of AR signaling has been demonstrated to be an important driver of progressive castration-resistant disease.^1^ We therefore assessed AR expression in pre- and post-treatment samples by immunohistochemistry and found it to be detectable and localized to the nucleus in the majority of persistent tumors, suggestive of ongoing activation (Fig 1C), although there was no difference in receptor staining or localization based on the level of treatment response (Fig 1D). A number of different mechanisms have been shown to reactivate AR signaling in castration-resistant disease. These include overexpression of the AR (frequently associated with *AR* amplification),^3^ which confers supersensitivity to subcastrate signaling androgen levels, the emergence of constitutively active receptor splice variants (in particular ARv7)^5^, mutations in the ligand-binding domain that permit promiscuous ligand binding, and an increase in intratumoral androgen synthesis.^16^ In addition, the presence of ARv7 in pretreatment samples has been reported to predict response to newer hormonal therapies such as abiraterone.^17^ However, we found no significant levels of signaling androgens in persistent tumors (Fig 1E), nor did we find an increase in the expression of the AR or its splice variants (Fig 1F and Data Supplement) or any mutations in *AR* coding regions, indicating that the mechanisms regulating castration persistence are distinct from those involved in castration resistance. In addition, ARv7 expression in pretreatment biopsy specimens was negligible compared with the full length receptor and did not predict treatment response (Data Supplement).

### Tumor Cells Surviving Acute Pathway Inhibition Demonstrate an Incomplete Epithelial-to-Mesenchymal Transition Phenotype

**FIG 2.**
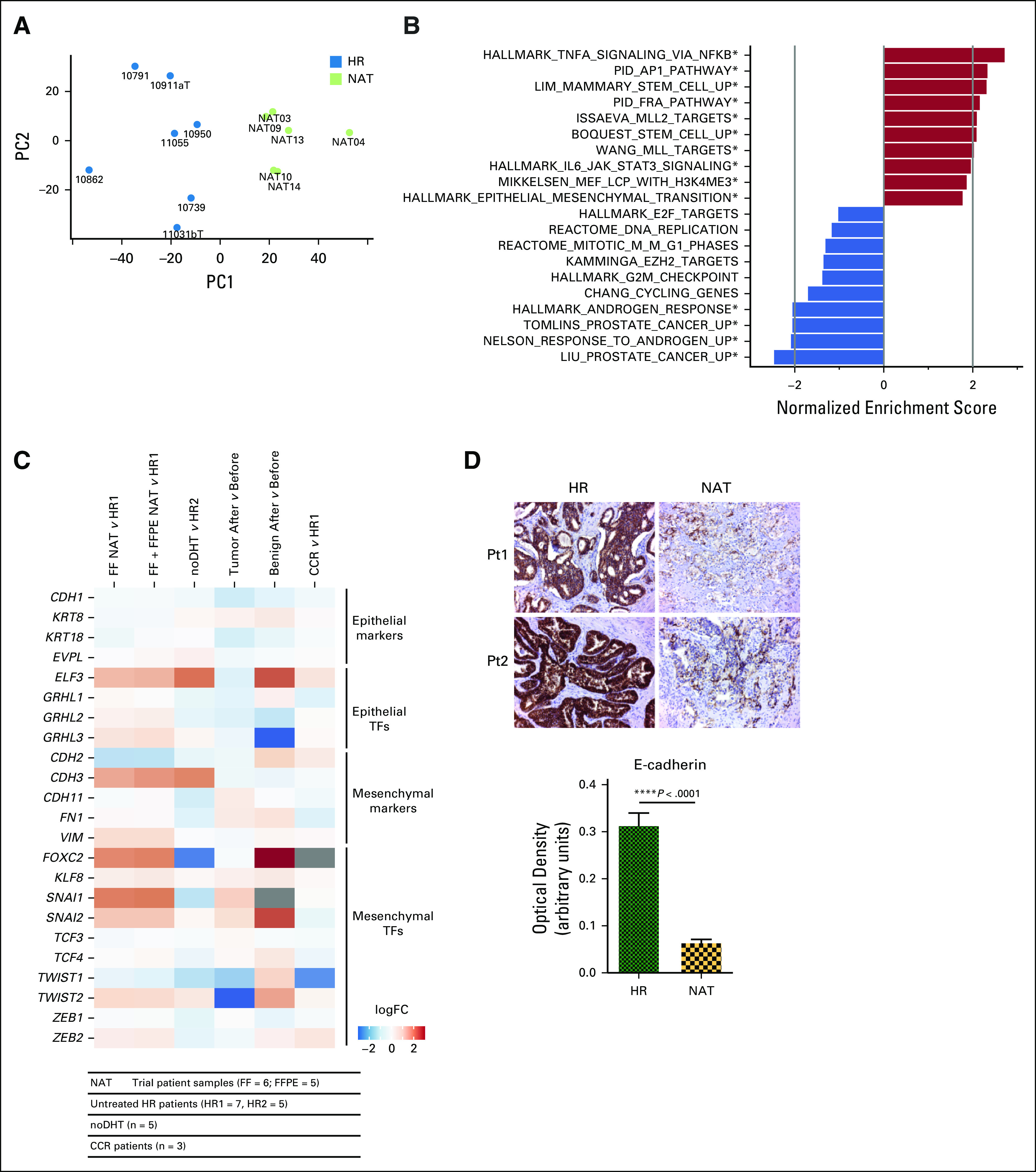
Tumor persistence is associated with global transcriptional reprogramming and phenotype switching. (A) Principal component analysis of global transcriptional profile reveals distinct clustering of NAT patients (n = 6 with histologically confirmed FF residual tumor tissue collected) compared with FF HR control (n = 7). (B) Normalized enrichment scores of selected gene sets up- or downregulated with neoadjuvant treatment from gene set enrichment analysis (asterisks indicate FDR < 0.05). (C) Heatmap demonstrating relative logfold change of selected epithelial-to-mesenchymal transition–related genes in various ADT-treated specimens compared with untreated controls. Gray indicates insufficient reads for differential expression. FFPE indicates microdissected FFPE tissue. Cohorts are described in detail in the Data Supplement. (D) Immunohistochemical analysis of E-cadherin expression in persistent tumors of NAT and untreated HR controls. Upper panel shows examples of staining from two patients in each group. Lower panel summarizes expression in HR controls (n = 5) and persistent NAT tumors (n = 12) as determined by digital image analysis. ADT, androgen deprivation therapy; CCR, clinical castration-resistant; FC, fold change; FDR, false discovery rate; FF, fresh-frozen; FFPE, formalin-fixed, paraffin-embedded; HR, high-risk; NAT, neoadjuvant therapy; noDHT, nontrial patients treated with ADT; PC1, principal component 1; PC2, principal component 2; Pt, patient; TF, transcription factor.

Accumulating evidence suggests that transcriptional reprogramming is a key event in acute resistance to therapy in tumor cells, usually associated with the emergence of a therapy-resistant phenotype.^18-22^ To probe this, we profiled global transcription in fresh-frozen residual tumors by RNA sequencing and observed clear differences in the transcriptional profile of resistant tumors compared with matched fresh-frozen untreated samples (Fig 2A; PC1 and PC2 capture 29.87% and 18.63% of the variance, respectively). Enrichment analysis revealed significant upregulation of gene sets associated with phenotype change, including stem-like and epithelial-to-mesenchymal transition (EMT), as well as genes regulated by chromatin modification (Fig 2B). Androgen-regulated genes were significantly downregulated, despite the presence and nuclear location of AR in persistent tumors, as were gene sets associated with cell proliferation (Fig 2B). We then interrogated the expression of key differentiation and regulatory genes in a number of complementary RNA-seq experiments (Fig 2C and Data Supplement) and found consistent upregulation of both markers of EMT and stemness in resistant tumor tissue. For EMT markers, similar levels of expression were observed in treated benign glands, but not in progressive castration-resistant samples, suggesting that this adaptive response is enriched in prostate epithelium surviving short-term AR pathway inhibition, although the number of CRPC samples analyzed is small. In contrast, upregulation of stem cell markers was observed in both disease states and in treated benign samples. To confirm that EMT was occurring, we measured the levels of the epithelial differentiation marker E-cadherin, loss of which is a key defining feature of EMT, and found consistent loss of expression in persistent tumors (Fig 2D). However, significant variability in the level of expression within individual cells was observed. Similar levels of E-cadherin downregulation were also identified in persistent luminal cells within benign glands (Data Supplement), which also expressed predominantly nuclear AR (Data Supplement), confirming that the adaptive response that permits epithelial survival is not cancer cell–specific. Despite high staining for the key mesenchymal marker vimentin in the stroma of persistent tumors, we observed no instance of epithelial staining, indicating that resistant tumors are in an intermediate EMT state (Data Supplement).^23^

### Lack of Clonal Change in Nonresponders Suggests Intrinsic Resistance

**FIG 3.**
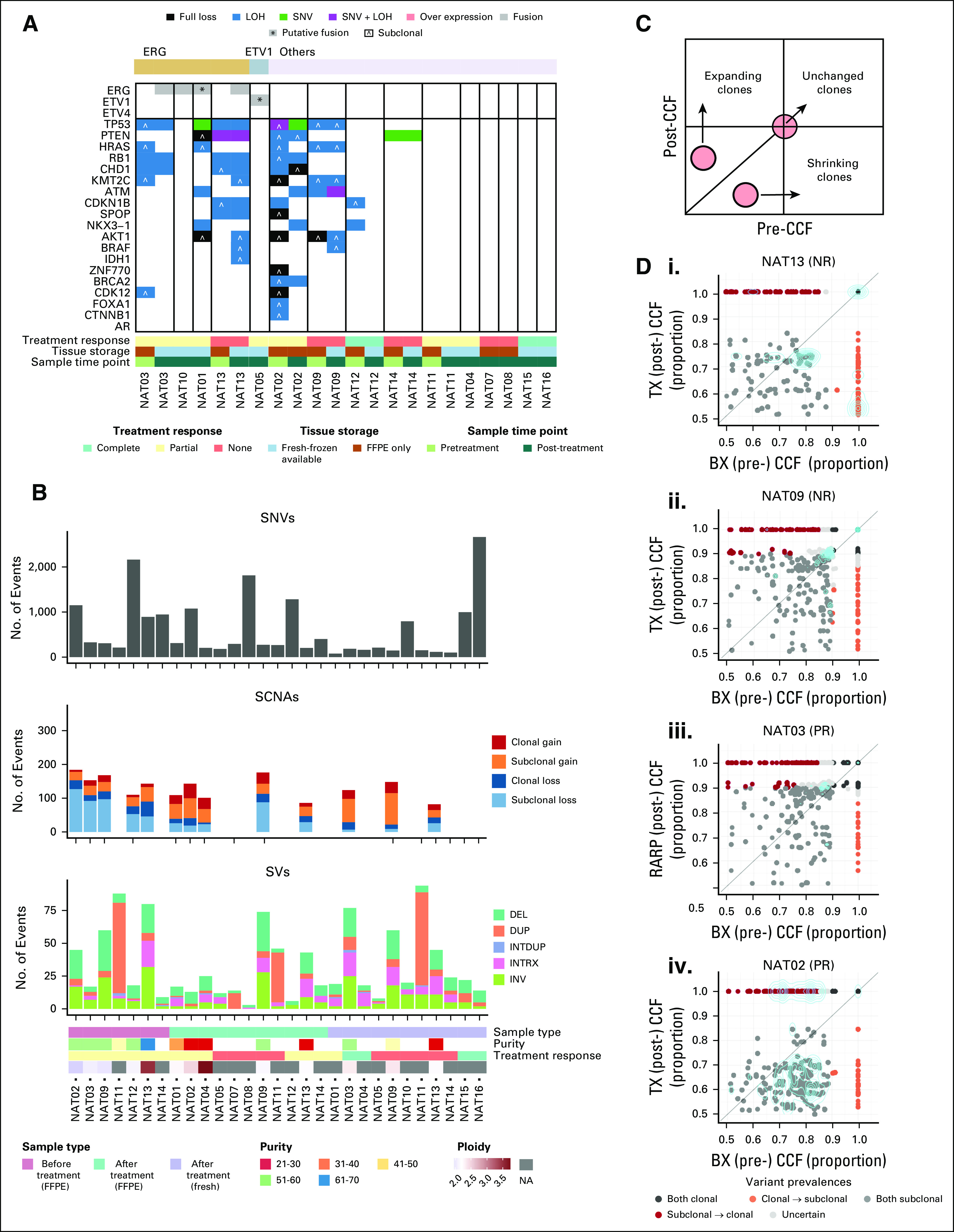
Persistence is not driven by somatic features or the evolution of a resistant clone. (A) Summary of somatic changes identified in NAT samples. Tumors are classified according to the molecular subtype present in any one sample from a patient. Subclonal indicates > 0.1 and < 0.9 copy number fraction from Battenberg.^57^ Subclonal copy number losses at < 0.1 CCF were only considered if the SCNA was present at > 0.1 in at least one other sample from the patient. Only losses were considered; no amplifications (> 4 allelic copies) affecting target genes were found. Samples with unclear purity and/or ploidy solutions were not considered for copy number losses. (B) Summary of SCNAs, SNVs, and SVs of samples. SCNAs are not displayed for samples with unclear purity and/or ploidy solutions. Subclonal SCNAs were considered as present at < 0.9 copy number fraction from Battenberg. (C) Schematic comparing CCFs in longitudinal samples, showing direction of clonal change. (D) Copy number fractions of segments at the same genomic location for pretreatment (BX) and post-treatment (TX [FFPE tissue] or RARP [fresh tissue]) samples from patients demonstrating varying levels of treatment response including NR, PR, and almost CR. The cyan lines indicate the 2D density distribution of the data. BX, biopsy (before treatment); CCF, cancer cell fraction; CR, complete response; DEL, deletion; DUP, duplication; FFPE, formalin-fixed paraffin-embedded; INRTX, inter-chromosomal translocation; INTDUP, interspersed duplication; INV, inversion; LOH, loss of heterozygosity; NA, not available; NAT, neoadjuvant therapy; NR, no response; PR, partial response; RARP, robot assisted radical prostatectomy; SCNA, somatic copy number aberration; SNV, singlenucleotide variant; SV, structural variant; TX, after treatment.

To investigate if this cell plasticity was related to the emergence of new driver lesions at the DNA level, we performed whole-genome sequencing calling single-nucleotide variants (SNVs), somatic copy number aberrations (SCNAs), and structural variants on paired pre- and post-treatment tumor samples (Figs 3A and 3B; Data Supplement). Successful sequencing was obtained from all residual tumors (n = 15, as one patient had no residual tumor), but only from 7 of 16 diagnostic specimens because of limited tumor tissue input and formalin-fixed paraffin-embedded (FFPE) induced DNA degradation. *ETS* fusions were identified in 5 of 15 patients, with the remaining patients falling into the Other category in The Cancer Genome Atlas.^24^ Although we did observe enrichment of specific genomic drivers with treatment in individual patients (for example, an *RB1* loss of heterozygosity event in NAT02), there was no consistent association between known drivers and tumor response, and in general, post-treatment samples had fewer detected somatic aberrations. Given that one potential mechanism of drug resistance is the clonal expansion of a distinct cell population intrinsically resistant to ADT,^25^ we performed clonal analysis with the PyClone algorithm^26^ using SNV and SCNA data across longitudinal patient samples (Figs 3C and 3D). We also performed an orthogonal clonal analysis by comparing SCNA segments in sample pairs. Of the seven patients with paired pre- and post-treatment WGS data, clonality was resolvable in four. To test for potential SNV artifacts in the FFPE samples, which may affect clonal analysis, we compared base pair transitions at different allele frequencies between matched fresh-frozen and FFPE samples. We observed no significant C>T/G>A enrichment in the 1%-10% or 10%-25% VAF ranges (*P* = .3352 and *P* = .7985, respectively, using a one-sided paired *t* test; Data Supplement). In the majority of patients, although evidence of subclones was identified in both pre- and post-treatment specimens, no significant changes in clonal makeup were identified in resistant tumors in patients NAT09 and NAT03 (Figs 3Di-3Diii and Data Supplement). NAT13 (partial responder) showed some evidence of a shrinking clone in the SCNA data (Data Supplement); however, this was not supported in the SNV analysis. In particular, no new genomic drivers were observed, indicating that tumor cell populations present before treatment were intrinsically resistant to ADT or had the intrinsic ability to adapt to the castrate state. In contrast, in one patient who demonstrated an almost complete pathologic response, we identified significant changes in the subclonal makeup, with evidence for the enrichment of a treatment-resistant subclone in both the SNV and SCNA data (Fig 3Div and Data Supplement).

### Pretreatment *SNAI2* Copy Number Status Correlates With Tumor Response

**FIG 4.**
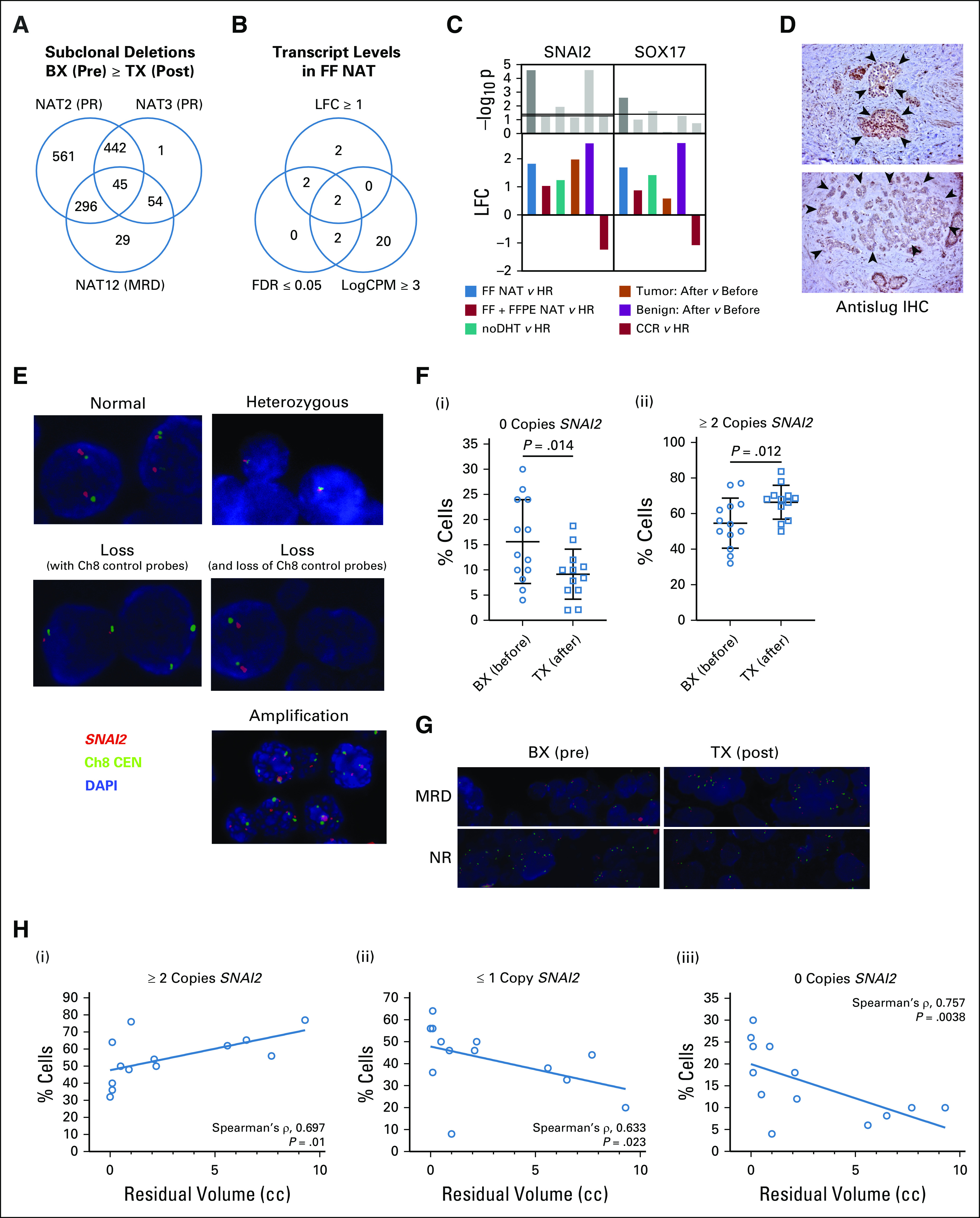
SNAI2 loss correlates with tumor response. (A) Venn diagram summarizing overlapping gene deletions identified subclonally in pretreatment samples but not in post-treatment specimens. (B) Prioritization of overlapping genes from (A) on the basis of level of expression in FF NAT versus HR RNA-seq data set. (C) Relative logfold change in *SNAI2* transcript measured by RNA-seq in various ADT-treated specimens compared with untreated controls. (D) Representative images (high power) of Slug immunohistochemical staining in residual tumors. Arrowheads indicate residual tumor foci. (E) Indicative fluorescent in situ hybridization images (magnification ×100) of tumor samples following hybridization with probes against *SNAI2* (red) and Ch8 centromere (green). (F) Mean ± standard deviation of percentage of cells with (i) deep deletion or (ii) ≥ 2 copies of *SNAI2* in before and after treatment specimens. (G) Representative images of changes in the prevalence of cells harboring deep deletions of *SNAI2* in pre- and post-treatment specimens in patients with MRD (NAT16) and NR (NAT11). (H) Scatter plots (i-iii) demonstrating the relationship between the prevalence of cells with the indicated *SNAI2* copy number state and residual tumor volume. ADT, androgen deprivation therapy; BX, biopsy (before treatment); CCR, cancer cell fraction; CPM, counts per million; DAPI, 4',6-diamidino-2-phenylindole; FDR, false discovery rate; FF, fresh-frozen; FFPE, formalin-fixed paraffin-embedded; HR, high-risk; IHC, immunohistochemistry; LFC, logfold change; MRD, minimal residual disease; NAT, neoadjuvant therapy; noDHT, nontrial patients treated with ADT; NR, no response; PR, partial response; TX, after treatment.

Given that SCNA events are more important in driving localized prostate cancer progression than SNVs and that copy number loss is more frequently observed than copy number gain,^24^ we reasoned that a copy number loss event would be the most likely somatic aberration defining treatment sensitivity. We therefore performed an overlap analysis of copy number loss events present subclonally in pretreatment specimens but not detectable in residual persistent tumors, focusing on the three patients with the best response (NAT2, NAT3, and NAT12) for whom paired pre- and post-treatment WGS data were available, identifying 45 candidate genes (Fig 4A). We reasoned that if loss of expression was important for treatment sensitivity, then overexpression might drive treatment resistance. We checked the expression levels of these 45 genes in matching RNA-seq data and found that only two were significantly overexpressed (logfold change > 1; FDR < 0.05) at reasonable transcript abundance (log counts per million > 3) (Fig 4B) and had consistent expression across the different RNA-seq data sets (Fig 4C). Unexpectedly, one of the prioritized genes was *SNAI2*, which encodes the transcription factor Slug, a key regulator of EMT that directly represses E-cadherin expression^27^ and is expressed in residual tumor epithelium (n = 13) (Fig 4D and Data Supplement). Using fluorescent in situ hybridization (FISH) to directly measure gene copy number at the cellular level (Fig 4E), we confirmed that treatment with profound ADT resulted in the loss of cells deficient for *SNAI2* (Figs 4F and 4G) and that the prevalence of *SNAI2*-deficient cells in pretreatment biopsy specimens correlated with tumor response (Fig 4H).

## DISCUSSION

We performed a phase II study of a novel combination of AR-targeting agents in patients with clinically localized prostate cancer at high risk of disease recurrence and, despite biochemical responses in all patients, observed significant variability in measured tumor response. Similar to previous studies of intense AR signaling inhibition,^15,28-30^ we found 25% of patients demonstrated complete response or near CR, with the remainder displaying either partial tumor regression or no histologic evidence of response. Despite AR being present and nuclear in the majority of patients with residual disease as previously observed,^15,28^ we did not identify a significant contribution of known AR-driven mechanisms, a key feature of progressive CRPC, to acute tumor persistence. In fact, transcriptional profiling indicated that AR signaling was significantly suppressed in residual tumors, consistent with previous findings.^31^

How treatment resistance emerges under the selective pressure of systemic therapy is a topic of intense speculation.^32^ Certainly, current evidence suggests that selection of subclones harboring genomic variants offering a pro-survival advantage is the predominant mechanism of prostate cancer persistence following intense AR signaling inhibition. For instance, in a whole-exome sequencing analysis of one or more tumor foci from 18 patients following 24 weeks of neoadjuvant leuprolide and abiraterone, Sowalsky et al^31^ identified enrichment of *RB1* loss in residual disease. Similarly, McKay et al^29^ recently reported an association between residual tumor volume and *PTEN* aberrations and *ERG* expression. Although we also observed enrichment for a small number of known drivers between pre- and post-treatment specimens, particularly at the subclonal level, we observed no consistent pattern across patients. However, given the well-recognized heterogeneity of localized prostate cancer,^33^ we interpret these results with caution.

An alternative explanation is that responding tumors harbor a high proportion of cells before treatment that are fatally sensitive to AR signaling inhibition. By analyzing pre- and post-treatment specimens, we found that resistance to short-term AR signaling inhibition is characterized by an adaptive drug-tolerant persister intermediate EMT state in residual tumor cells that echoes the phenotype changes described in vitro in diverse cell types,^18^ including human-derived prostate cancer cells.^34,35^ We observed that this phenotype switching is shared by benign prostate epithelium, suggesting that it is a default survival program under the hostile conditions of AR signaling inhibition, and by integrating data on genomic variants selectively lost with treatment with upregulated transcripts in residual malignant and benign glands identified *SNAI2* as a likely key regulator of this survival switch. In particular, we hypothesize that *SNAI2* deficiency in pretreatment tumor cells render them susceptible to cell death induced by AR signaling inhibition. Certainly, this concept would be consistent with recent observations regarding the importance of persistent luminal cells in the regeneration of the mouse prostate after castration-induced involution.^36^

Snai2 (previously known as Slug) is a prototypical EMT transcription factor that in addition to directly repressing E-cadherin expression has increasingly defined roles in stem cell maintenance, lineage commitment, and resistance to apoptosis.^37^ Overexpression of Snai2 is associated with a poor prognosis in multiple tumor types^38-41^ and specifically in metastatic prostate cancer cell lines has been shown to promote proliferation and invasiveness.^42,43^ Importantly, Snai2 expression protects tumors cells from induced cell death in response to a diverse array of therapies, including cytotoxic chemotherapy,^44,45^ radiation,^46,47^ and tyrosine kinase inhibitors.^48,49^ Within the prostate, Snai2 has been shown to be the key regulator of EMT in both benign and malignant epithelium,^50,51^ and as we also observed in multiple cohorts, expression is upregulated in clinical prostate cancers acutely treated with androgen deprivation,^52^ although this finding is not universal.^53^ Biallelic loss of *SNAI2* is likely to interfere with this EMT switch, preventing the emergence of ADT tolerance and eventual to tumor cell demise.

Complete clonal loss of *SNAI2* is uncommon in prostate cancer, being reported in 0.6% of localized cases in The Cancer Genome Atlas cohort.^24^ Similarly, in our analysis, *SNAI2* loss was only identified subclonally, and on FISH analysis, biallelic loss was only observed in up to a maximum of 30% of cells, even in extreme responders. Mechanisms other than genomic loss of the *SNAI2* locus may be relevant for hormone responsiveness as at least one patient had a good partial tumor response despite having only a very low level of single-copy loss or deep deletion of *SNAI2*. For instance, *SNAI2* expression is known to be regulated by epigenetic modification^54^ and microRNAs,^55^ two mechanisms we have not explored in this study. It is also likely that other EMT regulators are important (for instance ZEB2, which is also consistently overexpressed following AR signaling inhibition); however, on the basis of our integrated analysis, we prioritized *SNAI2* in this study. In addition, given the small number of samples included in the cohort, further validation in a larger cohort is required.

In conclusion, taken together, our data indicate that resistance to short-term AR signaling inhibition in prostate cancer is driven by molecular mechanisms that are distinct from those underpinning progressive castration-resistant outgrowth. Rather, tumor resistance is characterized by an adaptive drug-tolerant persister intermediate EMT state that is shared with benign prostate epithelium. These findings support the role of Slug as a key transcriptional regulator of these changes^51^ and are supported by animal models and limited human explant studies.^56^ We show that adaptation and survival appear to be the default state, with tumor regression being associated with the selective depletion of vulnerable cells defined by loss of *SNAI2*. These findings indicate that *SNAI2* has potential as a predictive biomarker of response to AR-targeting drugs and that agents targeting *SNAI2* expression would be expected to augment therapeutic response.

## Data Availability

Whole-genome and RNA sequencing data that support the findings of this study have been deposited in EGA with the primary accession code EGAS00001003172, data set accession EGAD00001006640.
